# Effectiveness of nurse-led intervention on health risks in perimenopausal women: A mixed-methods study

**DOI:** 10.6026/973206300212421

**Published:** 2025-08-31

**Authors:** Gnana Malar Periyakaruppan, Shankar Shanmugam Rajendran, Gomathy Priya Venkatachalam, Anitha Parasuraman, Muthupetchi Ramachandran, Gnana Sarjana Ramakrishnan, Malathi Dhakshnamoorthy

**Affiliations:** 1 Department of Community Health Nursing, College of Nursing, Madras Medical College, The Tamil Nadu Dr. MGR Medical University, Chennai, Tamil Nadu, India; 2 Department of Child Health Nursing, College of Nursing, Madras Medical College, The Tamil Nadu Dr. MGR Medical University, Chennai, Tamil Nadu, India; 3 Department of Medical Surgical Nursing, College of Nursing, Madras Medical College, The Tamil Nadu Dr. MGR Medical University, Chennai, Tamil Nadu, India

**Keywords:** Perimenopause, nurse-led interventions, health risk assessment, women's health, community care

## Abstract

The impact of nurse-led interventions on health risk assessments and healthcare challenges among perimenopausal women in Choolai, Chennai is of interest. A total of 60 women, aged 40-55, were divided into experimental and control groups. The experimental group showed a significant 13.01% reduction in health risk scores compared to the control group's 1.55% reduction. Qualitative analysis identified key themes including sleep issues, reliance on informal care and physical and emotional symptoms. The study highlights the effectiveness of community-based, nurse-led programs in improving health outcomes and addressing perimenopausal challenges, with implications for scalable healthcare frameworks.

## Background:

Perimenopause is a transitional phase before menopause, typically occurring between ages 40 and 50, marked by hormonal changes and various physical and psychological symptoms. This period, lasting 4 to 8 years, often reduces quality of life [1]. Around 75-80% of women experience symptoms such as hot flashes, night sweats and mood swings, with 30-40% reporting significant functional impairment [2]. Reduced estrogen increases the risk of osteoporosis, contributing to 20% of lifetime bone loss [3], while the risk of cardiovascular issues also rises, with 26.4% of women developing hypertension [4]. Additionally, 35% experience psychological challenges, including anxiety and depression [5]. Health risk assessments, covering symptoms like hot flashes, osteoporosis risk, and cognitive impairment, are crucial but often inaccessible due to healthcare awareness and training gaps, with 36.1% of women missing these screenings [6, 7]. Affordability is another barrier, as 45% of low-income women cannot afford essential tests [8]. Tailored health frameworks are vital, with nurse-led interventions showing promise. These interventions, including education, counseling, and lifestyle coaching, have proven effective. Around 40% of women lack awareness of perimenopausal health risks, but nurse-led education has improved symptom understanding and preventive behaviors in 85% of participants [9]. Mental health outcomes also improve, with 40% of women reporting reduced anxiety and depression after counselling [10]. Nurse-guided interventions have led to better cardiovascular health in 50.2% of women and improved weight control in 55.9%, reducing the risks of diabetes and hypertension [11, 12]. Qualitative data reveal that many women feel unprepared for perimenopause, with symptoms often misattributed to aging, delaying treatment [13, 14]. Financial constraints, particularly in rural and low-income areas, limit access to screening and treatment [15]. Global statistics highlight the scale of the issue, with 26% of the global female population over 50 in 2021 and over 1.2 billion women expected to be menopausal by 2030 [16]. The prevalence of symptoms is notably high among perimenopausal women in Asia and India. Therefore, it is of interest to describe the effect of nurse-led interventions on health risk assessment and explore healthcare challenges among perimenopausal women.

## Materials and Methods:

This study used a mixed-methods approach to explore the effectiveness of nurse-led interventions on health risk assessment and the healthcare challenges faced by perimenopausal women. The research design employed an explanatory sequential design, consisting of a quantitative phase followed by a qualitative phase. The quantitative part employed a quasi-experimental, non-randomized control group design, with 60 women (30 in the experimental group and 30 in the control group) participating. The experimental group received nurse-led interventions like education on perimenopausal symptom management, lifestyle modification, weight management, education and counselling, and support systems, while the control group received routine care. Health risks were assessed before and after the intervention using a researcher-developed tool. The qualitative part used a phenomenological design to explore women's experiences through unstructured interviews with 5 participants. The study was conducted in Choolai, Chennai, over a period of four weeks. The sample consisted of women aged 40-55 years who met the inclusion criteria. Non-probability convenience sampling was employed for the quantitative phase, while purposive sampling was used for the qualitative phase. The tools included a sociodemographic questionnaire, a clinical profile, and a 35-item Health Risk Assessment Tool with a scoring range of 0 to 105. Interviews were guided by six open-ended questions developed from the literature review. Ethical approval was obtained from Madras Medical College, Chennai -03 (IEC-MMC/Approval/34112024) and informed consent was taken from participants. Quantitative data were analyzed using SPSS version 22, with results shown as percentages, means, and chi-square tests. Data triangulation and saturation were ensured to enhance the study's credibility.

## Results:

This study aimed to assess the effect of nurse-led interventions on health risk assessment and to explore healthcare challenges among perimenopausal women. The mean age of the participants is 46.0 ±3.89 years. Most participants had no formal education (36.67% in the experimental group, 43.33% in the control group) and were married. Homemakers dominated the experimental group (60.00%), while self-employment was higher in the control group (46.67%). The experimental group mostly earned less than ₹20,000 (53.33%), whereas the control group earned ₹20,000-₹50,000 (60.00%). A majority in both groups were Hindus (80.00% experimental, 83.33% control) and lived in nuclear families. More experimental participants lived in rented homes (63.33%), while the control group mostly owned homes (56.67%). Non-vegetarian diets were common (73.33% experimental, 76.67% control). No significant differences were found between groups (p > 0.05). During the pretest, health risk assessments of perimenopausal women in both groups revealed similar symptom distributions for most variables, with no significant differences (p > 0.05). However, significant differences were observed in symptoms like night sweats (χ^2^=14.07, p=0.01), mood swings (χ^2^=17.19, p=0.01), and irritability (χ^2^=19.31, p=0.001), among others. The experimental group generally reported fewer or less severe symptoms compared to the control group. Notable differences also appeared in irregular periods, low sex drive, joint pain, weight gain, and osteoporosis. These findings suggest a baseline variation that may influence post-intervention outcomes. The experimental group had a mean health risk score of 33.00 (31.42%) before the test and 19.33 (18.41%) after the test. This indicates that their health risk decreased by 13.01%. The control group, on the other hand, had a mean pretest score of 34.80 (33.14%) and a post-test score of 33.17 (31.59%), representing a 1.63% drop. These data show that the experimental group experienced a significantly greater decrease in health risk compared to the control group (Table 1 - see PDF). Healthcare challenges among perimenopausal women were explored through three main themes. Theme 1: Well-being focused on sleep and irregularity. Fragmented, inconsistent sleep left women exhausted and vulnerable to anxiety, metabolic changes, and cardiovascular risks. One participant shared, "I have sleep issues" (P1). Irregular menstrual cycles, with unpredictable timing and fluctuating flow, created confusion and concern about when to seek advice. One participant noted, "Recently it doesn't come on time; sometimes less flow, sometimes heavy" (P1). Theme 2: Care examined family and hospital care. Relatives often recognized mood shifts, though occasional misunderstandings heightened stress. One participant mentioned, "Sometimes they understand, but sometimes they say I always get angry" (P4). Symptoms were typically managed at home or in small local clinics, with hospitals being reserved for severe, visible problems. "If the headache is heavy, then I go to the nearby one" (P1) explained another participant. Theme 3: Symptoms discussed pain and anger. Knee, back, and abdominal pain, linked to estrogen-related bone and muscle changes, significantly limited daily activities. One participant reported, "My lower abdomen hurts a lot" (P3). Hormonal fluctuations also reduced emotional tolerance, increasing irritation and disrupting social roles, with one participant noting, "Now, I get very angry" (P4). Significant associations were found between health assessment outcomes and sociodemographic variables, particularly education level (χ^2^ = 9.72, p = 0.01), where individuals with a high school education showed lower health risk. Employment status also showed significance (χ^2^ = 7.57, p = 0.02), with self-employed individuals at lower risk.

## Discussion:

This mixed-methods study demonstrates that most urban perimenopausal women initially begin in low-to-moderate health-risk categories, aligning with a survey by Ortmann *et al.* (2020) [17]. The absence of high-risk cases at baseline suggests that community screening and rising health literacy may already be mitigating extreme risk; yet, nearly half of the group still entered the moderate tier, underscoring an unmet need for targeted support. Nurse-led interventions resulted in a 13% absolute reduction in mean risk scores, significantly surpassing the 1.6% decline observed under routine care. Comparable magnitudes were achieved, where lifestyle education reduced the composite risk among postmenopausal women (Rathnayake *et al.* 2019) [18]. These findings confirm that nurses through personalized counseling, monitoring, and follow-up-are catalysts for sustained lifestyle change across diverse South Asian settings. Post-test analysis revealed that education, employment, normal Body Mass Index and moderate physical activity predicted lower risk only within the intervention group. These findings were supported by El Hajj *et al.* (2020) [19], who indicate that when nurses led guidance to personal resources, favourable socio-demographic traits amplify benefits, whereas routine care does not reduces health risk [19]. Practically, this supports stratifying counselling intensity by baseline education, occupation and weight status. Qualitative themes deepened these results. Poor sleep, cycle irregularity, joint pain and anger eroded well-being, aligned with the study by Refaeei *et al.* 2023 [13]. Women valued family empathy yet often self-managed or used local clinics, highlighting gaps in formal care pathways. Integrating family-centred education and streamlined referral channels could therefore enhance adherence and reach.

## Conclusion:

Nurse-led interventions improved the health of perimenopausal women by reducing health risks and managing symptoms like sleep issues and mood swings. Women who received tailored support had better outcomes than those with routine care. These findings highlight the importance of nurse-led programs in supporting women during perimenopause.
